# Clinical Outcomes in Low Risk Coronary Artery Disease Patients Treated with Different Limus-Based Drug-Eluting Stents - A Nationwide Retrospective Cohort Study Using Insurance Claims Database

**DOI:** 10.1371/journal.pone.0122860

**Published:** 2015-04-07

**Authors:** Chao-Lun Lai, Ching-Fen Wu, Raymond Nien-Chen Kuo, Yen-Yun Yang, Ming-Fong Chen, K. Arnold Chan, Mei-Shu Lai

**Affiliations:** 1 Department of Internal Medicine and Center for Critical Care Medicine, National Taiwan University Hospital Hsin-Chu Branch, Hsin-Chu, Taiwan; 2 Department of Internal Medicine, College of Medicine, National Taiwan University, Taipei, Taiwan; 3 Institute of Epidemiology and Preventive Medicine, College of Public Health, National Taiwan University, Taipei, Taiwan; 4 Department of Internal Medicine, Mennonite Christian Hospital, Hualien, Taiwan; 5 Institute of Health Policy and Management, College of Public Health, National Taiwan University, Taipei, Taiwan; 6 Center for Comparative Effectiveness Research, National Center of Excellence for Clinical Trial and Research, National Taiwan University Hospital, Taipei, Taiwan; 7 Department of Internal Medicine, National Taiwan University Hospital, Taipei, Taiwan; 8 Department of Medical Research, National Taiwan University Hospital, Taipei, Taiwan; 9 Graduate Institute of Oncology, College of Medicine, National Taiwan University, Taipei, Taiwan; Kaohsiung Chang Gung Memorial Hospital, TAIWAN

## Abstract

The clinical outcomes of different limus-based drug-eluting stents (DES) in a real-world setting have not been well defined. The aim of this study was to investigate the clinical outcomes of three different limus-based DES, namely sirolimus-eluting stent (SES), Endeavor zotarolimus-eluting stent (E-ZES) and everolimus-eluting stent (EES), using a national insurance claims database. We identified all patients who received implantation of single SES, E-ZES or EES between January 1, 2007 and December 31, 2009 from the National Health Insurance claims database, Taiwan. Follow-up was through December 31, 2011 for all selected clinical outcomes. The primary end-point was all-cause mortality. Secondary end-points included acute coronary events, heart failure needing hospitalization, and cerebrovascular disease. Cox regression model adjusting for baseline characteristics was used to compare the relative risks of different outcomes among the three different limus-based DES. Totally, 6584 patients were evaluated (n=2142 for SES, n=3445 for E-ZES, and n=997 for EES). After adjusting for baseline characteristics, we found no statistically significant difference in the risk of all-cause mortality in three DES groups (adjusted hazard ratio [HR]: 1.14, 95% confidence interval [CI]: 0.94-1.38, p=0.20 in E-ZES group compared with SES group; adjusted HR: 0.77, 95% CI: 0.54-1.10, p=0.15 in EES group compared with SES group). Similarly, we found no difference in the three stent groups in risks of acute coronary events, heart failure needing hospitalization, and cerebrovascular disease. In conclusion, we observed no difference in all-cause mortality, acute coronary events, heart failure needing hospitalization, and cerebrovascular disease in patients treated with SES, E-ZES, and EES in a real-world population-based setting in Taiwan.

## Introduction

Since the advent of percutaneous intervention, the goal of coronary interventional treatment has been maximizing both the safety and efficacy of coronary revascularization. Stent implantation has offered an effective method of treating coronary heart disease.[1,2] The introduction of the first-generation drug-eluting stents (DES), i.e. sirolimus-eluting stent (SES) and paclitaxel-eluting stent (PES), markedly reduced the need for repeated intervention compared with angioplasty alone or the use of bare-metal stents.[3–6] Notwithstanding, the effective inhibition of in-stent neointimal formation by the first-generation DES may delay re-endothelialization, leave stent struts as the nidus for late stent thrombosis and give rise to safety concern.[6–9] For the first-generation DES, SES is superior to PES in terms of a significant reduction of the risk of re-intervention and stent thrombosis.[3,10]

The second-generation DES may be better than the first-generation DES in several aspects, such as incorporating newer agents (such as zotarolimus and everolimus), using more biocompatible polymers, and thinner strut thickness to ameliorate stent flexibility and deliverability.[11] In addition, newer generation DES has been shown to improve endothelialization in animal models,[12] and reduce stent thrombosis rate in randomized clinical trials.[13,14]

Studies attempting to compare the efficacy and safety between different second-generation limus-based DES have yielded conflicting results.[15–18] 23Besides, external validation of findings of clinical trials in daily “real-world practice” has been limited and a comparison between different limus-based DES with real-world data within the whole population is of interest. The aim of this study was to investigate the short- (<1 year) and long-term (≥ 1 year) clinical outcomes of different limus-based DES, namely SES, Endeavor zotarolimus-eluting stent (E-ZES), and everolimus-eluting stent (EES) using a national insurance claims database.

## Materials and Methods

### Source of Data

This is a retrospective cohort study using the reimbursement database of the National Health Insurance (NHI) program in Taiwan. The Bureau of National Health Insurance (currently the National Health Insurance Administration, NHIA) implemented the compulsory universal NHI program in Taiwan since 1995. More than 98% of the total Taiwanese population of 23 million is covered by the program. All medical institutions contracted with the NHIA must submit standard computerized claims for reimbursement. Following the “sampling audit and payment” system, the claims from healthcare providers must receive administrative and professional reviews of the NHIA.[19] As a single-payer health insurance system, the NHIA invites medical experts and professional medical associations to set up unified guides for professional review. Professional medical associations in Taiwan also have developed clinical guidelines concerning specific medical issues such as hypertension,[20] ST-elevation myocardial infarction (MI),[21] and heart failure (HF).[22]

The NHI claims database contains complete claims history of diagnoses and procedures, provided as the International Classification of Diseases Ninth Revision Clinical Modification (ICD-9-CM) codes, and drugs dispensed for every beneficiary. The diagnoses and procedures associated with cases of acute MI in Taiwan NHI claims database have been validated recently.[23] In this study, patients’ medical information was extracted from the NHI claims database. The patients’ records were then linked to the Taiwan National Death Registry (NDR) by patients’ identification numbers to evaluate mortality outcomes. The accuracy of Taiwan NDR has also been validated previously.[24] To comply with data privacy regulations, personal identities were encrypted and all data were analyzed in a de-identified manner. The protocol for this study was approved by the Institutional Review Board of National Taiwan University Hospital, which waived requirement for informed consent.

### Study Population

**Fig 1:**
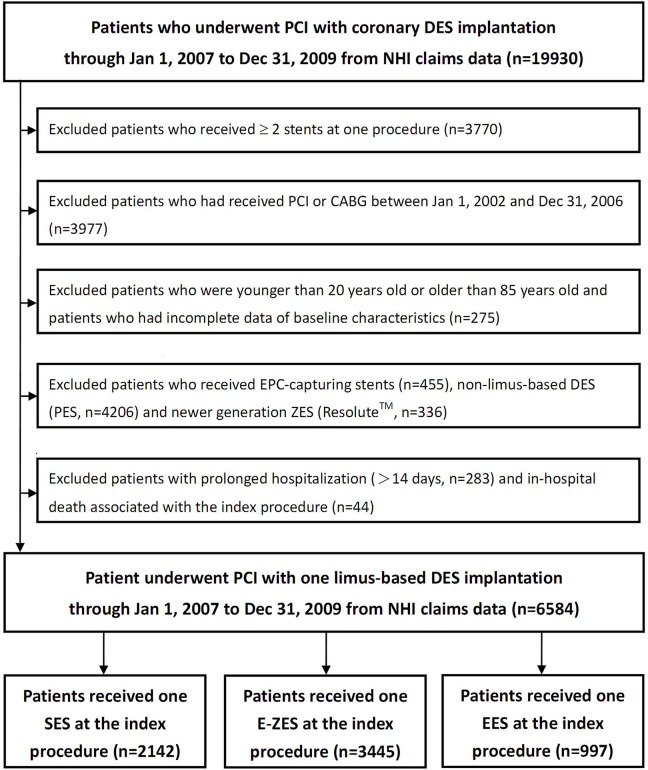
Flow chart of the study. Abbreviations: CABG: coronary artery bypass grafting; DES: drug-eluting stent; EES: everolimus-eluting stent; EPC: endogenous progenitor cell; E-ZES: Endeavor zotarolimus-eluting stent; NHI: National Health Insurance; PCI: percutaneous coronary intervention; PES: paclitaxel-eluting stent; SES: sirolimus-eluting stent; ZES: zotarolimus-eluting stent.

In Taiwan, SES has been reimbursed by the NHI program since December 2006, followed by E-ZES (April 2007) and EES (October 2008). We included all patients, aged between 20 and 85 years, who had insurance claims for one of the three limus-based DES (SES, E-ZES, and EES) from January 1, 2007 through December 31, 2009. The analyses were based on the type of stent implantation at the first recorded procedure (index procedure). The date of hospital discharge after the index procedure was operationally defined as the index date. Since we did not have the exact details of the length, size and location of the stents implanted, patients who received more than one stent during the index procedure were not included to ensure homogeneity of the study population. Patients who had ever received percutaneous coronary intervention (PCI) or coronary artery bypass grafting (CABG) within the 5-year period prior to the index procedure and patients with incomplete demographic or reimbursement data were excluded. Also, patients with complicated medical conditions, suggested by prolonged hospitalization (>14 days) associated with the index procedure were excluded. A flowchart for the identification of study subjects is shown.(Fig 1)

### Covariates

In addition to gender and age at index date, comorbidities were evaluated based on the NHI claims database within the 12-month baseline period prior to the index date. We determined the specific comorbid condition as the presence of corresponding diagnoses in no fewer than two out-patient records on different days or at least one in-patient record and coded as a binary variable. Previous MI or acute coronary syndrome (ACS) was defined as ICD-9-CM codes: 410–412. Besides, the comorbid conditions included in the Elixhauser comorbidity measurements (except acquired immune deficiency syndrome) were also evaluated accordingly.[25] Health care utilization of each patient was examined according to use of out-patient and in-patient services within the 12-month baseline period. Patients admitted through the emergency department in the index hospitalization were operationally defined as “ACS in the index procedure”. Among patients with “ACS in the index procedure”, we further classified those with ICD-9-CM codes: 410.0–410.6 and 410.8 as ST-elevation MI, those with ICD-9-CM codes: 410.7 and 410.9 as non-ST-elevation MI, and those with ICD-9-CM code: 411.X as non-MI ACS. Medications prescribed at hospital discharge after the index procedure, including angiotensin-converting-enzyme inhibitor (ACEi) or angiotensin receptor blocker (ARB), statin, beta-blocker, aspirin, clopidogrel, spironolactone, histamine-2 receptor blocker and proton pump inhibitor were identified.

### Follow-up and end-points

All the clinical outcomes were assessed through December 31, 2011. The primary end-point was all-cause mortality. Secondary end-points were composite end-point of acute coronary events including acute MI and emergency PCI, HF needing hospitalization, cerebrovascular disease, and composite end-point of repeated coronary revascularization including repeated PCI and CABG. Acute MI was defined as ICD-9-CM code of 410.X at hospital discharge. Emergency PCI was defined as hospitalization through emergency department with PCI during hospitalization. HF needing hospitalization was defined as discharge diagnosis of ICD-9-CM code of 428.X with use of intravenous loop diuretics (furosemide or bumetanide) during hospitalization. Cerebrovascular disease was defined as hospital discharge diagnosis of ICD-9-CM codes of 362.34, 430–436, 437.0–437.1, 437.9, 438, 781.4, 784.3, 997.0 with brain imaging such as computed tomography or magnetic resonance imaging performed during hospitalization.

### Statistical Analysis

For comparison of the baseline characteristics between the three DES groups, one-way ANOVA was used for continuous variables and the chi-square test was employed for categorical variables. Multivariate Cox proportional hazards regression model was used to estimate the relative hazards for developing different clinical end-points between different DES groups while controlling for age at index procedure, sex, year of index procedure, baseline comorbidities, health care utilization during the 12-month baseline period, ACS in the index procedure or not, and medications at discharge. The reference group comprised patients receiving SES. Kaplan-Meier survival curves were used to describe the difference in incidence of clinical end-points between patients who received different DES.

To account for the different clinical implications between short- and long-term outcomes of different DES, we also carried out time-varying Cox regression analyses with the relative hazards of developing clinical end-points within one year and after one year being estimated separately. The covariates adjusted in the time-varying Cox regression model were the same as in the conventional Cox proportional hazards regression model.

All the analyses were performed with SAS 9.3 software (SAS Institute Inc, Cary, North Carolina). A p-value<0.05 was considered statistically significant.

## Results

### Patient characteristics

From January 1, 2007 through December 31, 2009, 19930 patients receiving coronary DES implantation in Taiwan were identified in the NHI claims database. After the application of selection criteria, we identified 6584 patients who were treated with single limus-based DES of our interest: 2142 patients received SES, 3445 received E-ZES, and 997 received EES.(Fig 1) At baseline, more patients in the E-ZES group had renal failure while more patients in the SES group had metastatic cancer. The E-ZES group used more out-patient services than the other two groups. More patients in the SES group were admitted due to ST-elevation MI in the index procedure. As for the medications prescribed at discharge, patients in the EES group were more likely to have prescriptions of statins, and patients in the E-ZES group were more likely to have been prescribed with aspirin. Otherwise, the three groups did not differ significantly.(Table 1)

**Table 1:** Baseline characteristics of patients who received SES, E-ZES and EES.

	SES (n = 2142)		E-ZES (n = 3445)		EES (n = 997)		*P*
Age (years), mean (SD)	63.3	(11.7)	63.6	(11.4)	63.7	(11.2)	0.72
Male, n (%)	1610	(75.2)	2542	(73.8)	738	(74.0)	0.51
Procedure year, n (%)							<0.001
2007	623	(29.1)	612	(17.8)	0		
2008	838	(39.1)	1501	(43.6)	52	(5.2)	
2009	681	(31.8)	1332	(38.7)	945	(94.8)	
Comorbidities, n (%)							
Previous MI or ACS	421	(19.7)	698	(20.3)	183	(18.4)	0.41
Congestive Heart Failure	283	(13.2)	499	(14.5)	126	(12.6)	0.21
Cardiac arrhythmias	267	(12.5)	481	(14.0)	144	(14.4)	0.19
Valvular disease	127	(5.9)	232	(6.7)	77	(7.7)	0.16
Pulmonary circulation disorders	11	(0.5)	20	(0.6)	3	(0.3)	0.56
Peripheral vascular disorders	65	(3.0)	121	(3.5)	40	(4.0)	0.35
Hypertension	1441	(67.3)	2422	(70.3)	687	(68.9)	0.06
Paralysis	11	(0.5)	28	(0.8)	6	(0.6)	0.40
Other neurological disorders	34	(1.6)	59	(1.7)	22	(2.2)	0.46
Chronic pulmonary disease	325	(15.2)	544	(15.8)	162	(16.3)	0.71
Diabetes	769	(35.9)	1,261	(36.6)	359	(36.0)	0.85
Hypothyroidism	32	(1.5)	50	(1.5)	11	(1.1)	0.66
Renal failure	129	(6.0)	282	(8.2)	60	(6.0)	0.003
Liver Disease	61	(2.9)	100	(2.9)	34	(3.4)	0.66
Peptic ulcer disease, excluding bleeding	308	(14.4)	498	(14.5)	154	(15.5)	0.70
Lymphoma	3	(0.1)	8	(0.2)	-^a^		0.27
Metastatic cancer	10	(0.5)	6	(0.2)	-^a^		0.023
Solid tumor without metastasis	98	(4.6)	143	(4.2)	47	(4.7)	0.64
Rheumatoid arthritis / collagen vascular disease	99	(4.6)	136	(4.0)	38	(3.8)	0.40
Coagulopathy	3	(0.1)	13	(0.4)	-^a^		0.05
Obesity	18	(0.8)	23	(0.7)	7	(0.7)	0.76
Weight loss	16	(0.8)	16	(0.5)	4	(0.4)	0.30
Fluid and electrolyte disorders	36	(1.7)	86	(2.5)	23	(2.3)	0.13
Blood loss anemia	4	(0.2)	6	(0.2)	2	(0.2)	0.98
Deficiency anemia	16	(0.8)	32	(0.9)	10	(1.0)	0.70
Alcohol abuse	8	(0.4)	26	(0.8)	5	(0.5)	0.18
Drug abuse	-^a^		3	(0.1)	-^a^		0.25
Psychoses	7	(0.3)	9	(0.3)	2	(0.2)	0.80
Depression	79	(3.7)	135	(3.9)	46	(4.6)	0.46
Health care utilization							
Use of out-patient services, times (SD)	21.4	(16.4)	22.8	(17.5)	22.2	(16.9)	0.027
Use of in-patient services, times (SD)	0.4	(0.8)	0.4	(0.8)	0.3	(0.7)	0.18
Patients who ever used in-patient services, n (%)	558	(26.1)	953	(27.7)	257	(25.8)	0.30
ACS in the index procedure, n (%)							
ST-elevation MI	121	(5.7)	97	(2.8)	29	(2.9)	<0.001
Non-ST-elevation MI	100	(4.7)	129	(3.7)	30	(3.0)	0.06
Non-MI ACS	33	(1.5)	42	(1.2)	10	(1.0)	0.40
Medications at discharge, n (%)							
ACEi/ARB	1204	(56.2)	1925	(55.9)	549	(55.1)	0.83
Statin	1193	(55.7)	1896	(55.0)	594	(59.6)	0.038
Beta-blocker	1144	(53.4)	1927	(55.9)	524	(52.6)	0.07
Aspirin	1796	(83.9)	3017	(87.6)	846	(84.9)	<0.001
Clopidogrel	2083	(97.3)	3378	(98.1)	979	(98.2)	0.09
Spironolactone	76	(3.6)	163	(4.7)	42	(4.2)	0.10
H_2_-blocker or PPI	217	(10.1)	355	(10.3)	112	(11.2)	0.62

Abbreviations: ACEi: angotensin-converting-enzyme inhibitor; ACS: acute coronary syndrome, ARB: angiotensin receptor blocker; EES: everolimus-eluting stent; E-ZES: Endeavor zotarolimus-eluting stent; H_2_-blocker: histamine-2 receptor blocker; MI: myocardial infarction; PPI: proton pump inhibitor; SES: sirolimus-eluting stent; SD: standard deviation.

^a^In accordance with privacy regulations in Taiwan, the exact number of patients is not specified if it is less than 2.

### Primary end-point

**Fig 2:**
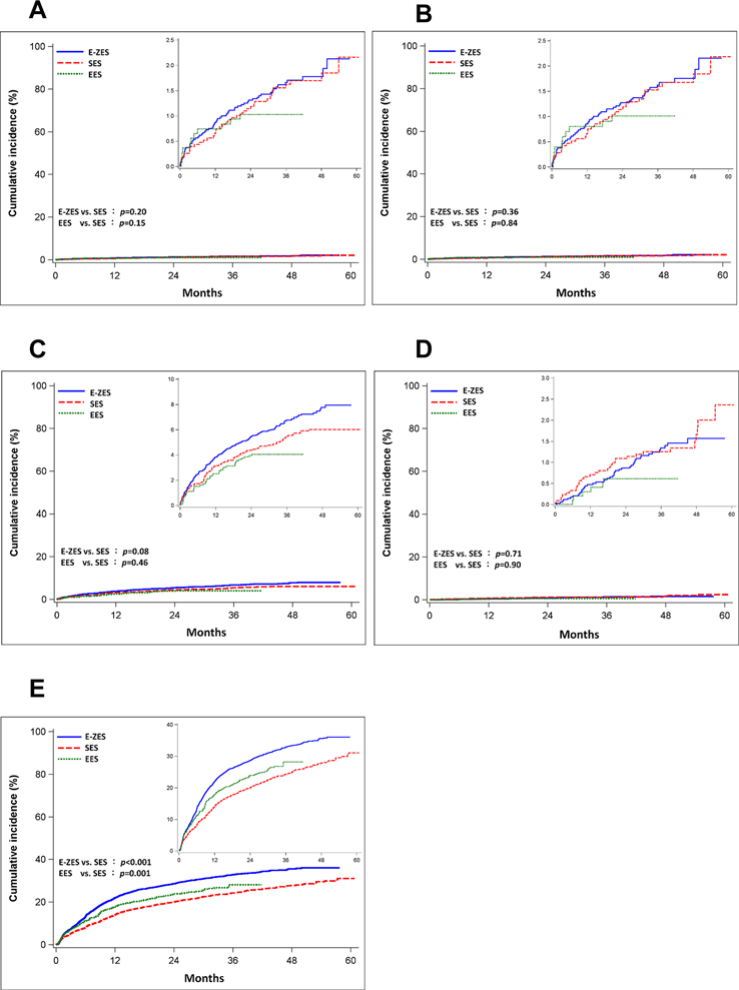
Kaplan-Meier cumulative incidences of different clinical end-points in three stent groups. All-cause mortality (Panel A), acute coronary events (Panel B), heart failure needing hospitalization (Panel C), cerebrovascular disease (Panel D), and repeated coronary revascularization (Panel E). Abbreviations: EES: everolimus-eluting stent; E-ZES: Endeavor zotarolimus-eluting stent; SES: sirolimus-eluting stent.

Till December 31, 2011, the median follow-up was 40.4 months in the SES group, 37.8 months in the E-ZES group and 28.9 months in the EES group. During follow-up, 495 patients died (162 in the SES group [7.6%], 294 in the E-ZES group [8.5%] and 39 in the EES group [3.9%]). After adjusting for baseline characteristics, we found no statistically significant difference in the risk of all-cause mortality in the three DES groups in spite of a trend of lower risk in EES group (adjusted hazard ratio [HR]: 1.14, 95% confidence interval [CI]: 0.94–1.38, p = 0.20 in E-ZES group compared with SES group; adjusted HR: 0.77, 95% CI: 0.54–1.10, p = 0.15 in EES group compared with SES group). The trend of mortality seemed to be consistent in both short- and long-term periods in the three DES groups.(Table 2 and Fig 2A)

**Table 2:** Clinical end-points of patients receiving SES, E-ZES and EES.

	Event no.	Follow-up time (PYs)	Incidence (per 1000 PYs)	Adjusted HR	95% CI	*p*
All-cause mortality						
SES	162	7203.8	22.5	1.00		
E-ZES	294	10860.0	27.1	1.14	0.94–1.38	0.20
within 1 year	103	3390.8	30.4	1.22	0.87–1.71	0.26
after 1 year	191	7469.2	25.6	1.10	0.87–1.39	0.43
EES	39	2400.2	16.2	0.77	0.54–1.10	0.15
within 1 year	16	988.6	16.2	0.74	0.42–1.30	0.30
after 1 year	23	1411.6	16.3	0.80	0.51–1.27	0.34
Composite end-point of acute coronary events						
SES	35	7130.3	4.9	1.00		
E-ZES	56	10755.2	5.2	1.22	0.80–1.88	0.36
within 1 year	29	3372.3	8.6	1.47	0.78–2.80	0.24
after 1 year	27	7382.9	3.7	1.05	0.59–1.87	0.88
EES	10	2382.9	4.2	0.93	0.46–1.90	0.84
within 1 year	8	982.3	8.1	1.49	0.62–3.56	0.37
after 1 year	2	1400.6	1.4	0.39	0.09–1.69	0.21
HF needing hospitalization						
SES	117	6984.6	16.8	1.00		
E-ZES	232	10501.6	22.1	1.22	0.97–1.53	0.08
within 1 year	131	3323.6	39.4	1.19	0.88–1.60	0.26
after 1 year	101	7178.0	14.1	1.27	0.90–1.78	0.17
EES	40	2346.9	17.0	0.87	0.61–1.25	0.46
within 1 year	25	975.0	25.6	0.83	0.52–1.31	0.42
after 1 year	15	1371.9	10.9	0.95	0.53–1.70	0.86
Cerebrovascular disease						
SES	32	7137.6	4.5	1.00		
E-ZES	45	10796.4	4.2	1.09	0.68–1.75	0.71
within 1 year	16	3384.2	4.7	0.77	0.38–1.58	0.48
after 1 year	29	7412.2	3.9	1.39	0.75–2.57	0.29
EES	6	2391.5	2.5	0.94	0.35–2.50	0.90
within 1 year	3	987.4	3.0	0.77	0.21–2.88	0.70
after 1 year	3	1404.1	2.1	1.08	0.29–4.03	0.90
Composite end-point of repeated coronary revascularization						
SES	542	5933.6	91.3	1.00		
E-ZES	1120	8218.4	136.3	1.43	1.29–1.58	<0.001
within 1 year	749	2967.2	252.4	1.66	1.45–1.90	<0.001
after 1 year	371	5251.2	70.7	1.13	0.96–1.33	0.14
EES	251	1984.5	126.5	1.32	1.11–1.55	0.001
within 1 year	177	884.7	200.1	1.50	1.23–1.83	<0.001
after 1 year	74	1099.8	67.3	1.07	0.82–1.41	0.61

Abbreviations: CI: confidence interval; EES: everolimus-eluting stent; E-ZES: Endeavor zotarolimus-eluting stent; HF: heart failure; HR: hazard ratio; PY: person-year; SES: sirolimus-eluting stent.

### Secondary end-points

As to the risk of composite end-point of acute coronary events, no difference was observed among the three groups (E-ZES vs. SES, adjusted HR: 1.22, 95% CI: 0.80–1.88, p = 0.36; EES vs. SES, adjusted HR: 0.93, 95% CI: 0.46–1.90, p = 0.84).(Table 2 and Fig 2B) Similarly, we found no difference in the three stent groups in risk of HF needing hospitalization (E-ZES vs. SES, adjusted HR: 1.22, 95% CI: 0.97–1.53, p = 0.08; EES vs. SES, adjusted HR: 0.87, 95% CI: 0.61–1.25, p = 0.46) (Table 2 and Fig 2C) and risk of cerebrovascular disease (E-ZES vs. SES, adjusted HR: 1.09, 95% CI: 0.68–1.75, p = 0.71; EES vs. SES, adjusted HR: 0.94, 95% CI: 0.35–2.50, p = 0.90).(Table 2 and Fig 2D) Both the E-ZES group and EES group, compared with SES group, had a higher rate of repeated coronary revascularization (E-ZES vs. SES, adjusted HR: 1.43, 95% CI: 1.29–1.58, p<0.001; EES vs. SES, adjusted HR: 1.32, 95% CI: 1.11–1.55, p = 0.001). The excessive risks existed only within the first year after stent implantation and disappeared after one year.(Table 2 and Fig 2E)

**Fig 3:**
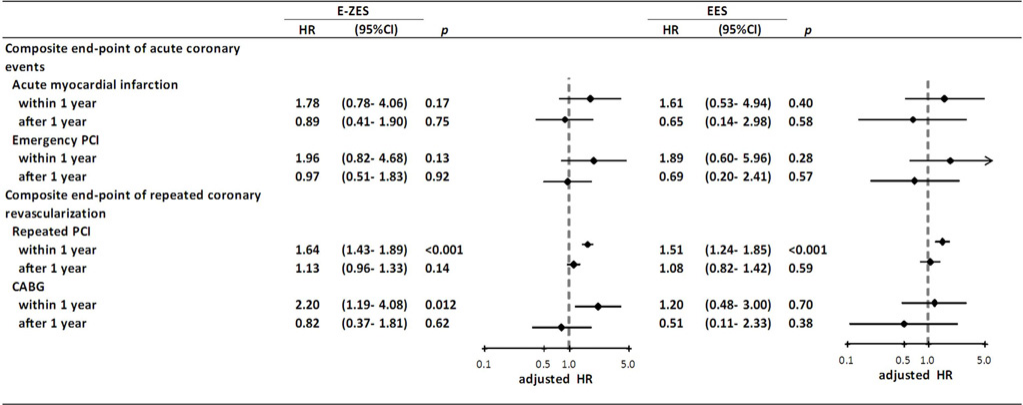
Relative risk of individual clinical end-point in two composite end-points among E-ZES and EES groups. The SES group was used as the reference. Abbreviations: CABG: coronary artery bypass grafting; CI: confidence interval; EES: everolimus-eluting stent; E-ZES: Endeavor zotarolimus-eluting stent; HR: hazard ratio; PCI: percutaneous coronary intervention; SES: sirolimus-eluting stent.

Considering individual clinical event in the composite end-point of repeated coronary revascularization, the E-ZES group was associated with higher rates of repeated PCI (adjusted HR: 1.64, 95% CI: 1.43–1.89, p<0.001) and CABG (adjusted HR: 2.20, 95% CI: 1.19–4.08, p = 0.012) within one year after stent implantation. The EES group had a higher rate of repeated PCI (adjusted HR: 1.51, 95% CI: 1.24–1.85, p<0.001) within one year compared with SES.(Fig 3)

## Discussion

In this study, we evaluated the clinical outcomes between three limus-based DES, namely SES, E-ZES, and EES, in a large real-world cohort of unselected, consecutive patients in Taiwan. We observed that there was no difference in risks of all-cause mortality, acute coronary events, HF needing hospitalization and cerebrovascular disease among these three DES groups. We only observed an increase in repeated coronary revascularization within the first year after stents implantation in both E-ZES group and EES group compared with SES group.

The SES had platform of stainless steel, with a strut thickness of 140 μm.[26] Second-generation DES, apart from incorporating new limus-based medications, have more biocompatible polymers, and are equipped with a cobalt or platinum chromium platform with thinner strut thickness of only 80–90 μm.[26] In animal models, second-generation DES had been shown to have better re-endothelialization.[12] Newer generation DES appear to preserve the anti-restenotic advantages, while mitigate the long-term risk of stent thrombosis;[27,28] thus represent a promising alternative to SES.

However, randomized clinical trials comparing E-ZES and SES have produced conflicting results. In the Randomized Controlled Trial of the Medtronic Endeavor Drug (ABT-578) Eluting Coronary Stent System Versus the Cypher Sirolimus-Eluting Coronary Stent System in De Novo Native Coronary Artery Lesions (ENDEAVOR III), 436 patients were randomized to E-ZES or SES in 3-to-1 ratio. Although E-ZES was associated with significantly higher late lumen loss and binary restenosis at 8-month angiographic follow-up compared with SES,[15] patients in the E-ZES group had lower risks of all-cause mortality, MI and major adverse cardiac events (MACE) than patients in the SES group at 5-year follow-up.[16] On the contrary, the Danish Organization for Clinical Trials with Clinical Outcome (SORT OUT III) trial, with randomization of 2332 patients to receive either the E-ZES or the SES, revealed higher risks of all-cause mortality, MI and target lesion revascularization in the E-ZES group at 18-month follow-up.[17] The differences in all-cause mortality and MI between E-ZES and SES groups attenuated after extended follow-up of 36 months but higher rate of target lesion revascularization in the E-ZES group remained.[18] Some studies found similar clinical efficacy between E-ZES and SES. For example, in the PROTECT (Patient Related OuTcomes with Endeavor versus Cypher stenting Trial), 8791 patients were randomized to receive either E-ZES or SES. At 3 years, rates of definite or probable stent thrombosis did not differ between groups.[29] Similarly, in the ZEST (Comparison of the Efficacy and Safety of Zotarolimus-Eluting Stent with Sirolimus-Eluting and PacliTaxel-Eluting Stent for Coronary Lesions) Randomized Trial, 2645 patients were randomized to E-ZES, SES and PES. At 12 months, the ZES group showed non-inferior rates of MACE compared with the SES group.[30] In a recently published network meta-analysis comparing clinical efficacy between different DES, all investigated DES were similar with regards to efficacy endpoints, except for E-ZES and PES, which were associated with a significant increase in the risks of target lesion and target vessel revascularization compared with other devices.[31]

EES has the thinnest strut (81μm) among the three limus-based DES evaluated in our study.[26] Therefore, better performance could be anticipated for coronary EES implantation. For instance, a total of 625 patients with acute MI were randomized (2:1) to receive EES or SES in the XAMI (XienceV Stent vs Cypher Stent in Primary PCI for Acute Myocardial Infarction) trial. The MACE rate was significantly lower for EES group compared with the SES group.[32] Besides, several clinical trials have found that EES performs equally to SES in specific clinical outcomes. The Scandinavian Organization for Randomized Trials with Clinical Outcome IV (SORT OUT IV) was an all-comer trial that randomized 2774 patients to receive either EES or SES. Both the EES and SES group had similar rates of primary composite end-point of cardiac death, MI, definite stent thrombosis and target vessel revascularization at 9-month, 18-month and 2-year follow-up.[33,34] In the RESET (Randomized Evaluation of Sirolimus-eluting versus Everolimus-eluting stent Trial), 3197 stable coronary artery disease Japanese patients were randomized to receive either EES or SES. Both clinical and angiographic outcomes after EES implantation were non-inferior to and not different from that after SES implantation after follow-up for one year.[35] Equally, in the EXCELLENT (Efficacy of Xience/Promus Versus Cypher to Reduce Late Loss After Stenting) Randomized Trial conducted in Korea, a total of 1443 patients undergoing PCI were randomized to receive EES or SES. The incidences of clinical end-points including target lesion failure and stent thrombosis at 9 months were not statistically different between the 2 groups.[36] Overall, a recent meta-analysis including 11 randomized trials (total 12869 patients) concluded that there was no significant difference regarding the risk of cardiac death or MI between EES and SES but a significant reduction in the risk of repeated revascularization in the EES arm was noted.[37]

In our study, we observed no significant difference regarding the risks of all-cause mortality, acute coronary events, HF needing hospitalization, and cerebrovascular disease in patients treated with SES, E-ZES, and EES. This result was consistent with the conclusions of the two previously mentioned meta-analyses[31,37] and provided strong support to the application of results of controlled clinical trials in real-world practice. However, we noticed a higher rate of repeated coronary revascularization which was driven principally by repeated PCI within the first year of stent implantation in both the E-ZES group and EES group compared with the SES group. This finding was different from the conclusion from the literature that E-ZES was associated with a significantly increase of target lesion and target vessel revascularization compared with SES[31] while EES was associated with a significant reduction in the risk of repeated coronary revascularization compared with SES.[37] Without procedural details of our study patients, we could not identify the objective of repeated PCI as scheduled PCI for non-target lesion/vessel in patients with multi-vessel disease or unintended PCI for target lesion/vessel failure. Therefore, repeated coronary revascularization could not be viewed as a true clinical event in our study and this issue needs further validation in the future.

### Limitations of the Study

Some limitations of the present study have to be acknowledged. Firstly, since the characteristics of lesions and details of procedures were not available in health insurance claims data, we excluded patients who received more than one stent suggestive of more complex coronary lesions to improve the comparability of the patients enrolled in this study. Therefore, our study represented results of simple, uncomplicated procedures in low risk coronary artery disease patients and could not be generalized to patients with complicated procedures. Secondly, although all three stent groups were highly similar with respect to baseline characteristics in our study, some clinical information such as left ventricular function, family history, body mass index and smoking status could not be obtained through the insurance claims data. Consequently, the present investigation is not immune to some of the inherent weaknesses of a retrospective cohort study such as the effect of residual confounding. Thirdly, the definitions of covariates and outcomes were based on ICD-9-CM codes. Accordingly, the accuracy and consistency of the data depended heavily on the training and expertise of coders which might vary among different hospitals. Finally, stent thrombosis could not be evaluated directly in this study although we included acute MI and emergency PCI as components of a secondary end-point.

## Conclusions

In a real-world population-based setting in Taiwan, we observed no difference in all-cause mortality, acute coronary events, HF needing hospitalization, and cerebrovascular disease in low risk coronary artery disease patients treated with SES, E-ZES, and EES. Hence, a coronary stent with a lower price may be the preference while performing DES implantation in low risk coronary artery disease patients from the perspective of health economics.
